# Single-Cell Transcriptomic Profiling Reveals KRAS/TP53-Driven Neutrophil Reprogramming in Luad: A Multi-Gene Prognostic Model and Therapeutic Targeting of RHOV

**DOI:** 10.32604/or.2025.062584

**Published:** 2025-05-29

**Authors:** Yinghui Ye, Yulou Luo, Yutian Sun, Yujie Zhang, Jiaxin Lin, Ziling Yang, Anping Xu, Bei Xue

**Affiliations:** 1 Department of Laboratory Medicine, Xinhua Hospital, Shenzhen, 518000, China; 2 Department of Breast Surgery, Affiliated Tumor Hospital of Xinjiang Medical University, Urumqi, 830000, China; 3 Department of Medical Oncology, Sichuan Cancer Hospital and Institute, Sichuan Cancer Center, School of Medicine, University of Electronic Science and Technology of China, Chengdu, 610000, China; 4 National Cancer Center/National Clinical Research Center for Cancer/Cancer Hospital & Shenzhen Hospital, Chinese Academy of Medical Sciences and Peking Union Medical College, Shenzhen, 518000, China; 5 Medical College, Shantou University, Shantou, 515000, China; 6 Department of Laboratory Medicine, Shenzhen Hospital, Peking University, Shenzhen, 518000, China

**Keywords:** Lung adenocarcinoma (LUAD), KRAS/TP53 mutations, neutrophil, prognosis, tumor microenvironment, single-cell analysis

## Abstract

**Objectives:**

The tumorigenic progression of Lung adenocarcinoma (LUAD), the predominant NSCLC subtype, is predominantly driven by co-occurring mutations in KRAS proto-oncogene (KRAS)/Tumor protein p53 (TP53). However, their impact on tumor microenvironment (TME) heterogeneity, particularly neutrophil dynamics, remains poorly understood. This present study aims to elucidate how KRAS/TP53 mutations reprogram the TME and develop a neutrophil-centric prognostic signature for LUAD.

**Methods:**

Leveraging single-cell RNA sequencing data and transcriptome data, neutrophil subpopulations were identified using Seurat and CellChat R packages, with trajectory analysis via Monocle2 R package. High-dimensional weighted gene co-expression network analysis (hdWGCNA), univariate Cox regression, and least absolute shrinkage and selection operator (LASSO) regression analyses were employed to generate a prognostic signature. Functional validation included Ras homolog family member V (RHOV) knockdown in A549/H1299 cells using siRNA, were assessed by cell counting kit 8 (CCK8) assay, wound healing assay, and transwell assay.

**Results:**

KRAS/TP53-mutated LUAD exhibited increased neutrophil infiltration, particularly IS MUT subtypes with enhanced OSM/CALCR/IL-1 signaling. A five-gene prognostic signature (MS4A1, ANLN, FAM83A, RHOV, KRT6A) stratified patients into high- and low-risk groups with divergent overall survival in the TCGA-LUAD cohort (*p* < 0.0001). AUCs achieved 0.73, 0.70, and 0.66 at 1-, 3-, and 5-year, respectively. External validation in immunotherapy cohorts (IMvigor210, GSE78220) confirmed the fine predictive capability of the prognostic signature in predicting treatment response. An integrated prognostic nomogram combining clinicopathological features and risk score further improved its clinical utility. Pseudotime analysis found that RHOV was essential for the growth of lung epithelial cells. RHOV knockdown significantly reduced the proliferation, migration, and invasion capabilities of A549/H1299 cells *in vitro*.

**Conclusion:**

KRAS/TP53 mutations may drive neutrophil heterogeneity in the TME of LUAD, addressing prognostic and therapeutic value. The five-gene signature and RHOV targeting offer translational relevance for risk stratification and therapy. These findings bridge genomic alterations with TME remodeling, advancing precision oncology in LUAD.

## 1 Introduction

As revealed by global cancer statistics in 2022, the incidence and mortality rates of lung cancer remain alarmingly high [1]. Lung adenocarcinoma (LUAD) represents the most common category of non-small cell lung cancer (NSCLC), comprising around 50% of all lung cancer instances [2]. Consequently, the overall disease burden of LUAD is concerning, particularly in light of the escalating severity of drug resistance, thus significant breakthroughs are essential to improve the overall prognosis [3]. The KRAS proto-oncogene (KRAS) and the Tumor protein p53 (TP53) are well-established tumor suppressor genes, and their mutations act as crucial drivers in the emergence and progression of various cancer types [4,5]. KRAS plays a pivotal role in signaling pathways downstream of the receptor tyrosine kinase family. In most cells, KRAS exists in an inactive state, but once activated, it can trigger multiple downstream signaling cascades, including the Mitogen-activated protein kinase (MAPK) pathway, the Phosphatidylinositol-4,5-bisphosphate 3 kinase (PI3K) pathway, and the Ral guanine nucleotide exchange factor (RalGEF) pathway [6]. These signaling pathways are integral to promoting cell survival, proliferation, and cytokine release. Conversely, TP53 is crucial for DNA damage repair, enabling cells with genetic errors during replication to undergo apoptosis, hence preventing these cells from continuing to divide and inhibiting the accrual of erroneous DNA information. However, in lung cancer treatment, further exploration is warranted due to the intrinsic challenges posed by targeting these two genes.

The tumor microenvironment (TME) is a comprehensive concept that denotes a dynamic and evolving complex composed of tumor cells, immune cells, fibroblasts, stromal cells, and extracellular matrix components [7]. These elements collectively influence the biological behavior of tumors through intricate and diverse signaling pathways [8,9]. Resistance to radiochemotherapy may also be associated with abnormal signaling interactions among these components [10]. Recently, substantial advances in omics analyses have significantly facilitated the dissection of TME composition and heterogeneity, providing multifaceted insights into TME and its relevant networks. Diverse pivotal regulators affecting TME heterogeneity and tumor-stroma interactions have been delineated from the perspectives of metabolic and senescence characteristics [11,12], the novel phenomena of disulfidptosis or cuproptosis [13,14], immunomodulatory features [15,16], and inflammatory characteristics [17]. However, the influence of gene mutations, particularly KRAS/TP53 mutations, on alterations in cellular composition within the downstream TME remains unclear. Therefore, we systematically dissected the TME traits of LUAD with KRAS/TP53 mutations and explore the related prognostic signature based on single-cell RNA sequencing (scRNA-seq) and transcriptome data, attempting to uncover actionable vulnerabilities for implementing precision oncology strategies and delineate therapeutic prioritization for mutation-specific LUAD.

## 2 Materials and Methods

### 2.1 Data Collection

GSE136246, GSE68465, GSE3141, GSE31210, GSE37745, GSE50081, and GSE78220 datasets were accessed from the Gene Expression Omnibus (GEO) database (www.ncbi.nlm.nih.gov/geo (accessed on 1 January 2025)). Bulk data for LUAD were acquired from The Cancer Genome Atlas (TCGA) database (https://portal.gdc.cancer.gov (accessed on 1 January 2025)). Additionally, transcriptome data for LUAD patients receiving immunotherapy were obtained from the IMvigor210 cohort. Samples that were duplicated or lacked complete survival and clinicopathological information were excluded from further analysis.

### 2.2 Single-Cell Analysis and Intercellular Communication Analysis

We utilized the Seurat R package to integrate the single-cell RNA sequencing data, applying the following filter criteria: nCount RNA ≥ 1000; nFeature RNA ≥ 200 & nFeature RNA ≤10,000; percent.mt (mitochondrial count) ≤20; percent.ribo (ribosomal count) ≤20. To normalize the data, we employed the LogNormalize method. Marker genes and hypervariable genes for each cell cluster were identified using the FindAllMarkers and FindVariableFeatures functions, respectively. Cell types were delineated according to classical molecule markers, and the UMAP method was utilized for visualization. Using CellChat R package’s programming tools, we measured both the quantity of cell signaling pairs and how actively different cell types interact through these molecular messages.

### 2.3 Single-Cell Trajectory Analysis

To uncover potential neutrophil subtypes arising from KRAS/TP53 mutations, we aimed to delineate their developmental pathways and gene expression dynamics through single-cell trajectory analysis. Concurrently, we sought to chart the evolution of epithelial cells within the LUAD microenvironment characterized by these mutations to identify early therapeutic targets. The Monocle2 R package was applied to analyze the evolutionary trajectory of neutrophils and epithelial cells in LUAD patients. Pseudo-time analysis was applied to explore the dynamics of gene expression patterns during the evolution of these cell types.

### 2.4 High-Dimensional Weighted Gene Co-Expression Network Analysis

High-dimensional weighted gene co-expression network analysis (hdWGCNA) enhances the dissection of scRNA-seq data, specializing in the management of high-dimensional datasets. Superseding traditional WGCNA, it effectively navigates vast gene expression data, yielding in-depth insights into cellular heterogeneity and enriching our understanding of gene expression through rigorous gene network construction. It excels at detecting subtle expression differences in single-cell data, demonstrating superior sensitivity and precision. Thus, we looked into the key features of genetic pattern of neutrophils using the hdWGCNA R package, which facilitated the identification of genes highly associated with neutrophils.

### 2.5 Consensus Clustering Analysis and Differential Analysis

Consensus clustering of the TCGA-LUAD cohort based on genes identified through hdWGCNA was analyzed by the ConsensusClusterPlus R package. The vaviable *k* represented cluster numbers, and the optimum *k* value was identified by cumulative distribution function (CDF). limma R package, a functional programmed tool for recognization of differentially expressed genes (DEGs), were applied between different clusters. Genes with *p* < 0.05 and |Log_2_FoldChange| > 0.5 were labeled DEGs. All identified DEGs underwent Gene Ontology (GO) and Kyoto Encyclopedia of Genes and Genomes (KEGG) functional enrichment analyses using the clusterProfiler and org.Hs.eg.db R packages.

### 2.6 Immune Microenvironment Dissection

Based on the TCGA-LUAD cohort, we quantified and compared immune infiltration levels between clusters using single-sample Gene Set Enrichment Analysis (ssGSEA). The StromalScore, ImmuneScore, and ESTIMATEScore were quantified and compared across clusters using the ESTIMATE algorithm. Additionally, the differential expression patterns of immune checkpoints were analyzed. The potential for tumor immune escape was quantified and compared among clusters using the Tumor Immune Dysfunction and Exclusion (TIDE) algorithm.

### 2.7 Establishment and Verification of the Prognostic Signature

Least Absolute Shrinkage and Selection Operator (LASSO) regression analysis was utilized to construct the prognostic signature based on DEGs identified in the TCGA-LUAD cohort. A risk score for each patient was calculated, and patients were subsequently divided into high-risk and low-risk groups according to the median risk score. Kaplan-Meier analysis was performed using the survminer R package to compare overall survival (OS) between the high-risk and low-risk groups. Additionally, time-dependent receiver operating characteristic (ROC) analysis was conducted using the timeROC R package, with the area under the curve (AUC) employed to evaluate predictive efficacy. Internal validation cohorts included the GSE37745, GSE3141, GSE50081, GSE68465, and GSE31210 datasets. The efficacy of the prognostic signature in predicting responsiveness to anti-Programmed cell death 1 (PD-1) therapy was externally verified in GSE78220 and IMvigor210 datasets. Atezolizumab was administered in the IMvigor210 dataset, while pembrolizumab and nivolumab were utilized in the GSE78220 dataset.

### 2.8 Exploration of Associations between Risk Score and Clinicopathological Features

Risk score across several clinical characteristics were compared. Univariate and multivariate Cox regression analyses were conducted to find prognostic effector. Furthermore, a predictive nomogram was developed using the ggpubr R package. Calibration curves, decision curve analysis, and AUC were employed to systematically assess the performance of the nomogram.

### 2.9 Identification of the Crucial Gene for Lung Epithelial Cell Survival

Scoring algorithms (AUCell, UCell, sing score, ssGSEA, and Add) were utilized to quantify the prognostic gene set. The scale function in R was employed to normalize the calculated gene set scores (from zero to one). This normalization involved centering the mean of each feature to zero by subtracting the mean value from every data point, followed by dividing the centered data by its standard deviation to scale the standard deviation of each feature to unity. This technique ensured the comparability of gene set scores derived from different algorithms during subsequent comparative analyses. The sum of corresponding scores was calculated, referred to as the “Scoring”. HighRisk and LowRisk subtypes of epithelial cells were classified according to the median score. Besides, Monocle R package was applied to fit evolutionary trajectory of lung epithelial cells and to indicate expression patterns of the five prognostic genes in pseudo-time.

### 2.10 Cell Source and Culture

Two human NSCLC cell lines, A549 (Procell, CL-0016, Wuhan, China) and H1299 (Procell, CL-0165), were selected for further experiments. Both cell lines were cultured in Roswell Park Memorial Institute-1640 (RPMI-1640) medium (Procell, PM150110B) supplemented with 10% fetal bovine serum (FBS) and 1% antibiotic solution. To ensure the integrity of our experimental data, all cell lines used in this study were rigorously tested for mycoplasma contamination. Mycoplasma tests were conducted using the PCR-based Mycoplasma Detection Kit (MedChemExpress, HY-K0552, Shanghai, China) prior to the initiation of experiments and periodically thereafter, following the manufacturer’s instructions. The absence of mycoplasma contamination in all cultured cells was confirmed before conducting any experiments.

### 2.11 siRNA Transfection

Small interfering RNA (siRNA) were transfected to cells. The siRNA sequences targeting Ras homolog family member V (RHOV) are as follows: siRNA1: Sense (SS) sequence: GGACGAUGUCAACGUACUAAU, Antisense (AS) sequence: UAGUACGUUGACAUCGUCCCU; siRNA2: SS sequence: GGCUGGAGAAGAAACUGAAUG, AS sequence: UUCAGUUUCUUCUCCAGCCGG. The sequence of the negative control siRNA (si-NC) is as follows: SS sequence: UUCUCCGAACG, AS sequence: UGUCACGUTT. Transfection was performed using Lipofectamine 3000 (Invitrogen, L3000015, Shanghai, China) according to the manufacturer’s instructions.

### 2.12 Western Blot Assay

Cells were subjected to lysis using a lysis buffer (Beyotime, R0046, Shanghai, China), and protein concentration was assessed utilizing the bicinchoninic acid (BCA) assay. The supernatant containing extracted proteins was separated by denaturing 10% sodium dodecyl sulfate-polyacrylamide gel electrophoresis (SDS-PAGE) and subsequently transferred to polyvinylidene fluoride (PVDF) membranes (Beyotime, P0965). Following a blocking step with 5% bovine serum albumin (BSA) (Beyotime, ST2249) for 2 h at room temperature, the membrane was incubated overnight at 4°C with the primary antibodies (RHOV: Abcam, ab54558, 1:200; β-actin: Abcam, ab8227, 1:1000, Shanghai, China). After this incubation, membranes were washed three times and then treated with goat anti-rabbit secondary antibody (Abcam, ab205718, 1:10,000). The images were captured using an Odyssey infrared imaging scanner (LI-COR, Tucson, AZ, USA).

### 2.13 Cell Counting Kit 8 (CCK8) Assay

Cell was plated in a 96-well plate with 100 μL of culture medium at a density of 5 × 10^4^ cells/well. Cell proliferative capability was evaluated at 0, 1, 2, 3, 4, and 5 days post-transfection. 10 μL of CCK8 solution (Beyotime, C0042) was added to each well, and the plate was incubated for 2 h at 37°C. Absorbance at 450 nm was then measured using a microplate reader (Molecular Devices, SpectraMax Plus 384, Shanghai, China).

### 2.14 Wound Healing Assay

Cells were grown in a 6-well plate (Beyotime, FCP060) until reaching 100% confluence. A linear scratch was created in the cell monolayer using a 200 μL pipette tip. The wells were then rinsed with serum-free medium to eliminate any detached cells and debris, followed by the addition of fresh medium. Images of the scratched region were captured using an inverted microscope (×100) (Fisher Scientific, LMI3PH2, Waltham, MA, USA) at 0, 12, and 24 h.

### 2.15 Transwell Assay

The transwell chamber (Corning, CLS3412, Shanghai, China) was coated with Matrigel (Corning, 356234). Cells were seeded in the upper chambers with FBS-free medium, while the lower chamber contained medium supplemented with 10% FBS. After a 24-h incubation at 37°C, non-invading cells were removed with a cotton swab. Cells that migrated to the bottom of the chamber were fixed with 4% methanol at 37°C for 10 min and stained with 0.1% crystal violet for 15 min. An inverted microscope (×200) (Fisher Scientific, LMI3PH2) was employed to count the invading cells in three randomly selected fields.

### 2.16 Statistical Analysis

All experiments were conducted with a minimum of three repetitions. Statistical analyses of *in vitro* experimental data and bioinformatic data were analyzed using R 4.0.3. Survival curves were compared using Cox regression analysis. The Wilcoxon rank sum test was employed to evaluate differences in expression levels between groups. Correlation analysis was conducted using the Pearson correlation coefficient, where |*r*| > 0.1 was considered relevant and *p* < 0.05 was considered statistically significant. Throughout the study, * indicates *p* < 0.05, ** indicates *p* < 0.01, *** indicates *p* < 0.001, and **** indicates *p* < 0.0001.

## 3 Results

### 3.1 Neutrophils Were More Abundant in the TME of MUT LUAD

**Figure 1:**
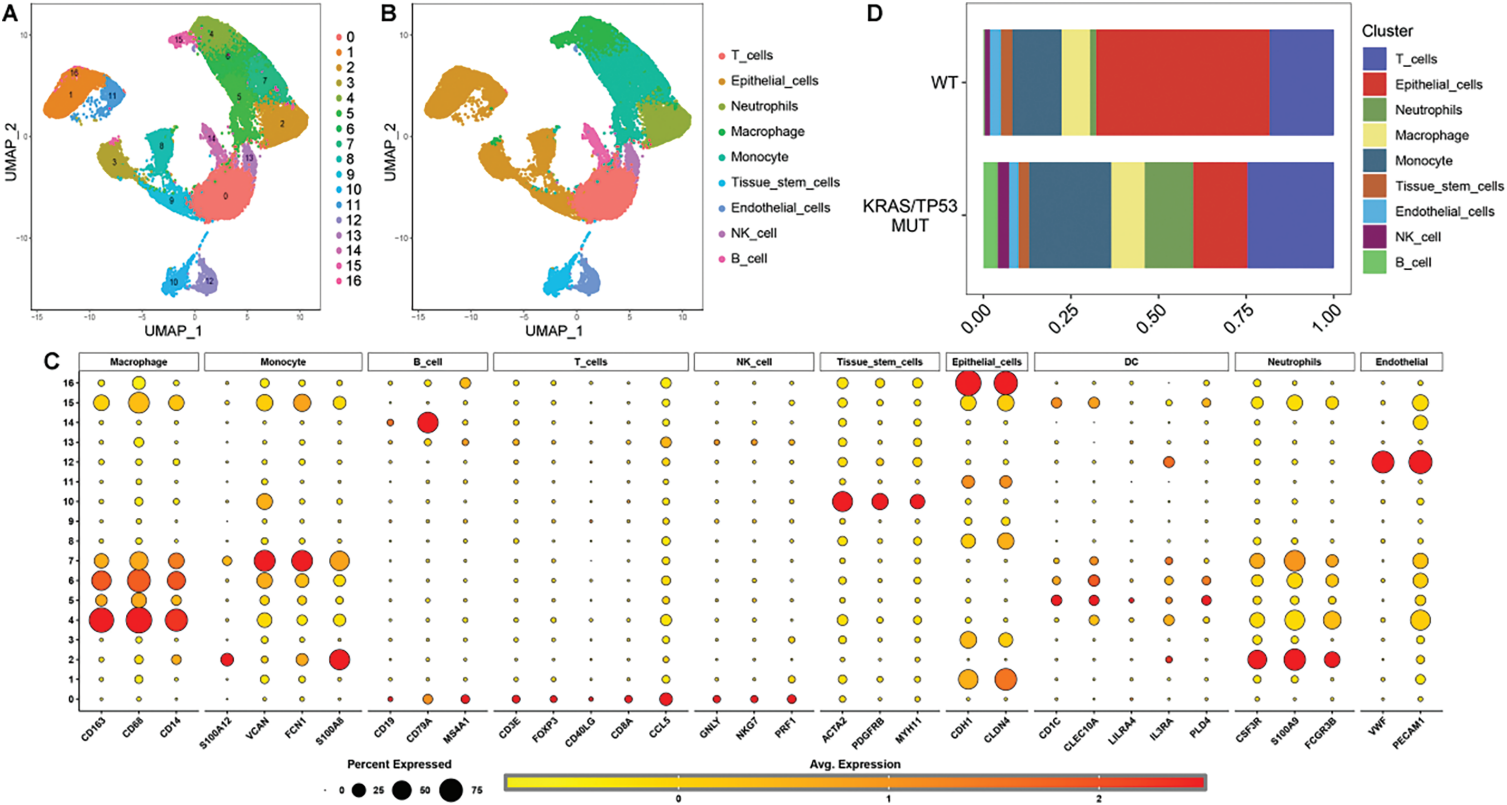
Cell type analysis and classification in KRAS/TP53 mutation. (**A**) UMAP diagram illustrating the results of dimensionality reduction analysis. (**B**) UMAP diagram depicting nine identified cell types: T cells, Epithelial cells, Neutrophils, Macrophages, Monocytes, Tissue stem cells, Endothelial cells, NK cells, and B cells. (**C**) Dot plot displaying the average expression of marker genes across 17 cell clusters. (**D**) Proportions of the nine cell types in the KRAS/TP53 MUT group compared to the WT group

We obtained 17 distinct clusters of cells through UMAP analysis (Fig. 1A). These 17 clusters of cells were subsequently categorized into nine cell types based on classical gene markers (Fig. 1B,C). Notably, we observed a pronounced abundance of neutrophils in the TME of LUAD with KRAS/TP53 mutations (MUT LUAD) (Fig. 1D).

### 3.2 IS MUT Neutrophil Was Identified as a Novel Neutrophil Subpopulation in LUAD

**Figure 2:**
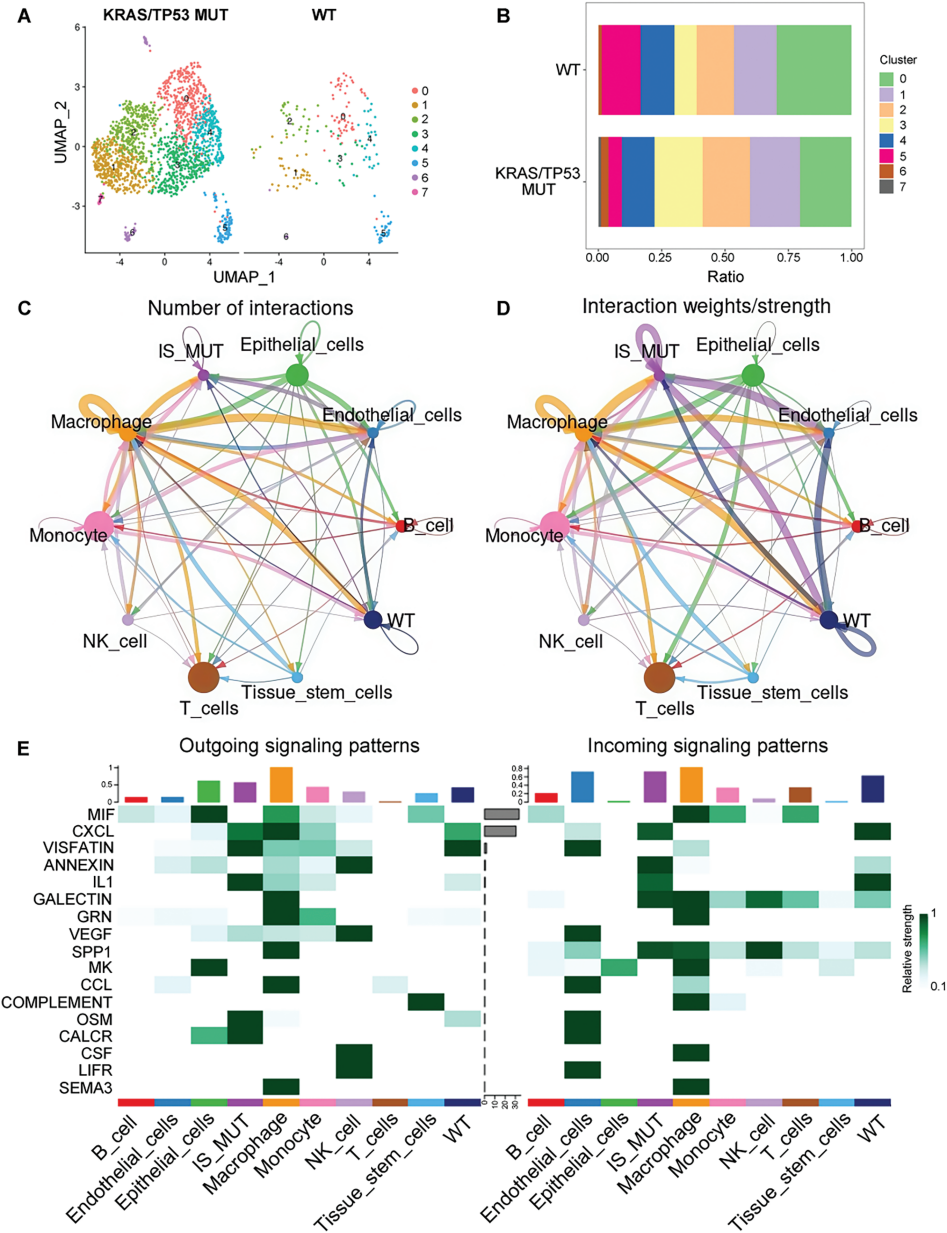
Neutrophil subpopulations and interactions in KRAS/TP53 mutation context. (**A**) UMAP diagram showing the distribution of eight neutrophil subpopulations in the KRAS/TP53 MUT and WT groups. (**B**) Proportions of the eight neutrophil subpopulations in the KRAS/TP53 MUT group *vs*. the WT group. (**C,D**) Number of interactions and interaction weights/strength for each cell type. (**E**) Heatmap illustrating the outgoing and incoming signal patterns of each cell type

**Figure 3:**
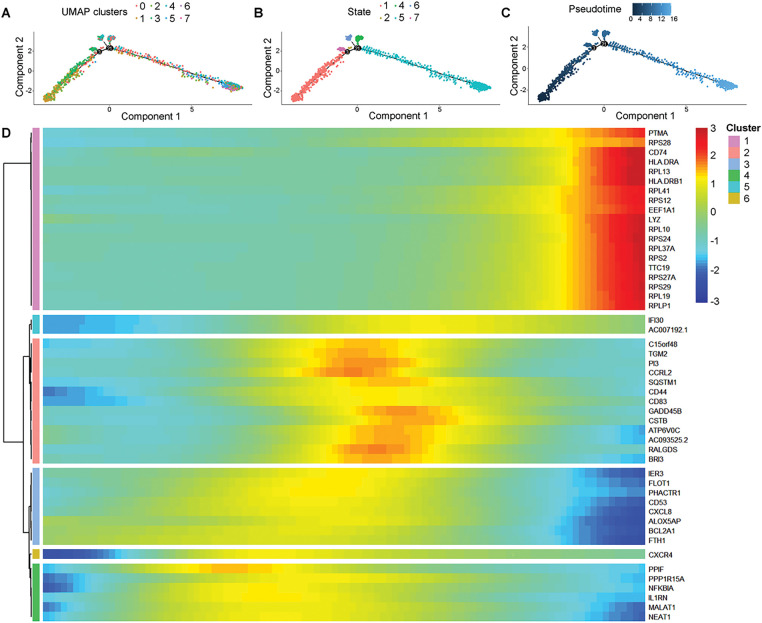
Pseudotime analysis of gene evolution in IS MUT cells within the KRAS/TP53 MUT group. (**A–C**) Trajectories depicting pseudo-time-dependent cellular states of IS MUT in the KRAS/TP53 MUT group. (**D**) The heatmap showing the evolution of 50 genes over pseudotime

We identified eight neutrophil subpopulations (Fig. 2A). Compared with the wild-type (WT) group, subpopulations 3, 6, and 7 exhibited a higher proportion in the KRAS/TP53 MUT group (Fig. 2B). Thereafter, we defined subpopulations 3, 6, and 7 as IS MUT neutrophils, while the remaining subpopulations were designated as WT neutrophils. The term “IS MUT” specifically refers to the collective of these three subpopulations (3, 6, and 7) rather than a broader characteristic driven by KRAS/TP53 mutations. Furthermore, the communication among B cells, neutrophils, endothelial cells, macrophages, and epithelial cells was notably robust during carcinogenesis (Fig. 2C,D). Analysis of intercellular signaling revealed that the interaction strength of IS MUT neutrophils was greater than that of WT neutrophils, particularly for signals such as Oncostatin M (OSM), Calcitonin receptor (CALCR), and Interleukin 1 (IL-1) (Fig. 2E). Additionally, three states of IS MUT neutrophils were delineated as pseudo-time progressed (Fig. 3A–C). A heat map illustrated the gene expression patterns during the evolution of neutrophils as pseudo-time changed (Fig. 3D).

### 3.3 Genes Related to IS MUT Neutrophils Were Indicated via hdWGCNA

**Figure 4:**
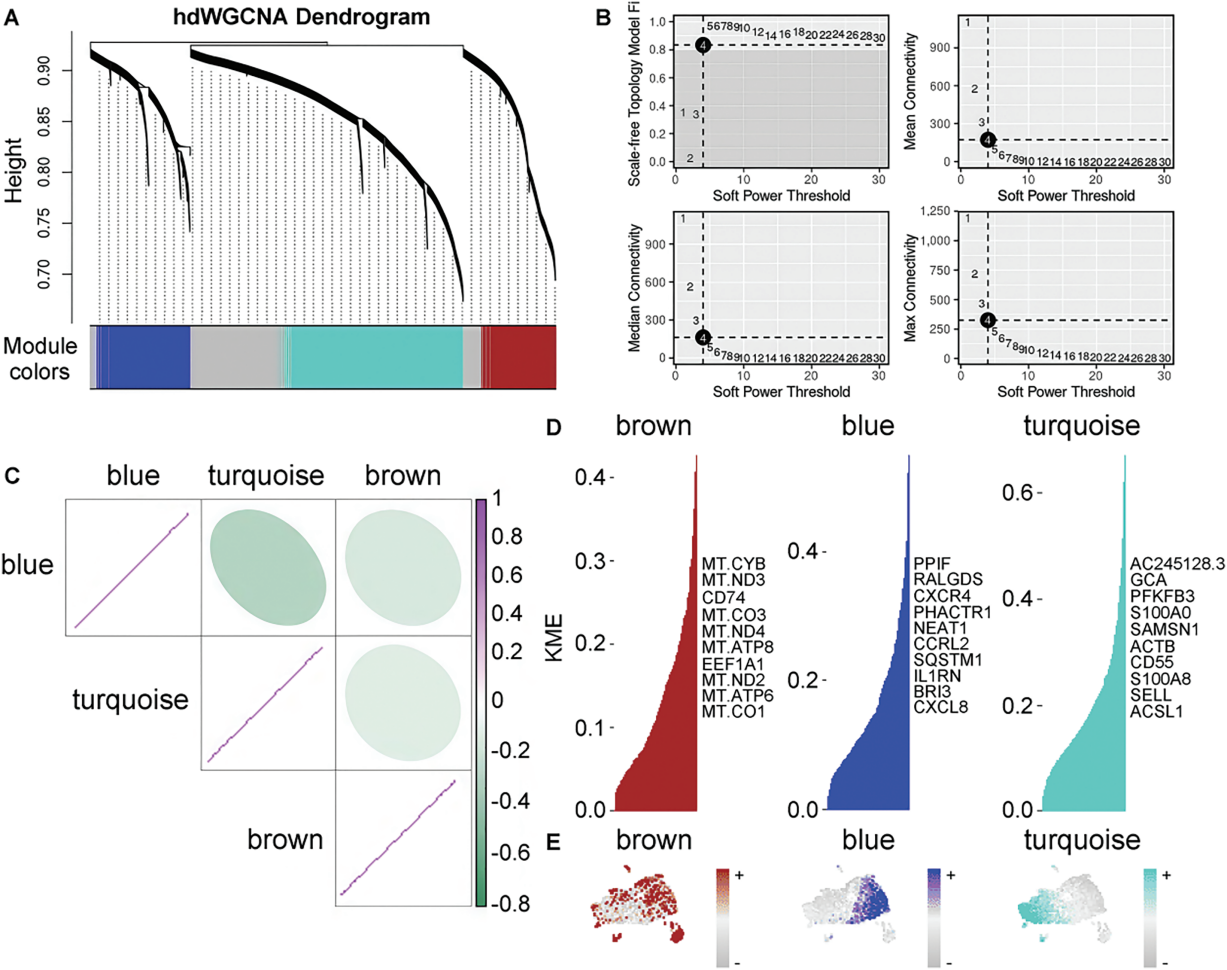
Gene module analysis and correlation in neutrophils with KRAS/TP53 mutation. (**A**) Hierarchical dendrogram from the hdWGCNA. (**B**) Optimal soft thresholds selected, displaying maximum, median, and average connectivity. (**C**) Heatmap demonstrating the correlation analysis between the three gene modules. (**D**) Three gene modules were identified according to standard processes, with the top 10 hub genes presented. (**E**) Distribution of the three gene modules in neutrophils within the KRAS/TP53 MUT group

A hierarchical hdWGCNA dendrogram was constructed, revealing three gene modules (Fig. 4A). At an optimum soft threshold of 4, the co-expression network was generated (Fig. 4B). Correlations within three identified gene modules were analyzed (Fig. 4C). Hub genes from the three gene modules were also determined (Fig. 4D), with the distribution of these modules observed in neutrophils (Fig. 4E). Notably, the blue and brown modules predominantly encompassed neutrophil subpopulations 3, 6, and 7 (Figs. 2A and 4E). Hence, our focus was directed towards the blue and brown gene modules.

### 3.4 Patients in C1 Harbored Better Prognosis Due to Active TME

**Figure 5:**
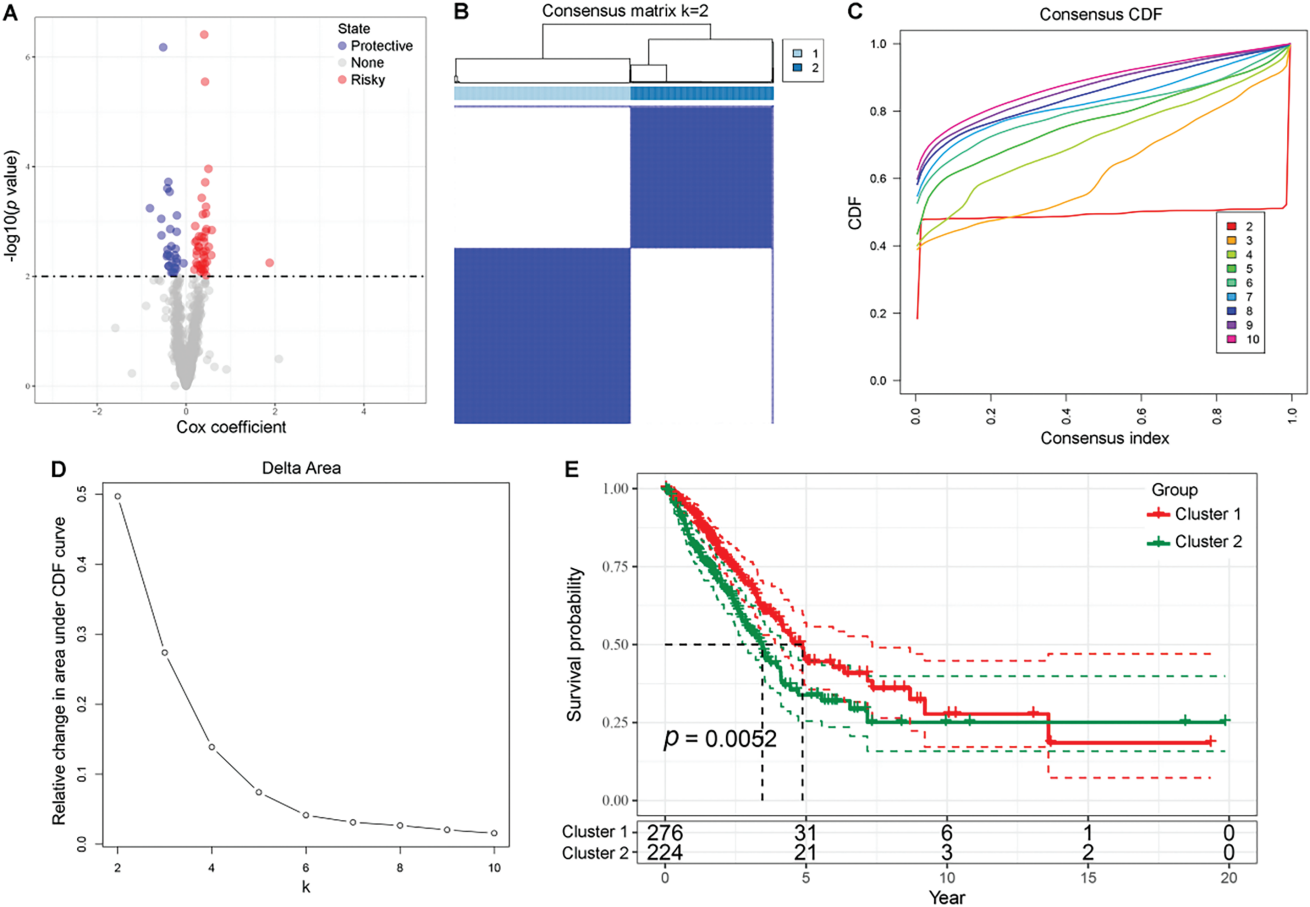
Univariate regression analysis and survival outcomes in LUAD patient clusters. (**A**) Volcano plot presenting the results of univariate regression analysis, with red indicating risky genes, blue indicating protective genes, and gray indicating genes with no significant difference. (**B**) Matrix heatmap showing LUAD patients divided into two clusters (*k* = 2). (**C,D**) The cumulative distribution function (**CDF**) is displayed in the delta plot and cumulative distribution curve plot. (**E**) Kaplan-Meier curve illustrating OS for two LUAD clusters

We identified 70 candidate genes from 773 genes in the blue and brown modules via univariate analysis (Fig. 5A). Two clusters, C1 and C2, were divided in the TCGA-LUAD cohort (Fig. 5B,D). Patients in cluster C1 exhibited prolonged survival probabilities compared to counterparts in cluster C2 (Fig. 5E). More abundant infiltration of immune cells were observed in the TME of C1 (Supplementary Fig. S1A). Utilizing the ESTIMATE algorithm, we found that the stromal and immune components of C1 were significantly higher than C2 (Supplementary Fig. S1B). Furthermore, compared to C2, the expression levels of immune checkpoints were significantly elevated in C1 (Supplementary Fig. S1C). TIDE, exclusion, and MDSC scores of C1 were significantly lower than those of C2 (Supplementary Fig. S1D,G,I), indicating that C2 exhibited a stronger immune escape capability. Additionally, dysfunction and IFNG scores of C1 were significantly higher than those of C2, potentially related to T cell exhaustion (Supplementary Fig. S1E,F). In summary, the heterogeneity of the immune microenvironment may serve as a driving factor contributing to the poorer prognosis observed in C2.

### 3.5 A Five-Gene Prognostic Signature Was Constructed and Well-Validated

494 DEGs were found between cluster C1 and C2 (Supplementary Fig. S2A). The DEGs were significantly enriched in pathways related to immune cell regulation and cell adhesion. Additionally, the DEGs primarily consisted of cell membrane components, such as endoplasmic reticulum and Major histocompatibility complex (MHC) proteins. They were also involved in molecular functions, including chemokine activation and immune receptor-ligand binding (Supplementary Fig. S2B,C). Results from functional enrichment analyses indicated that the identified DEGs related to immune responses and the invasion or metastasis of tumor cells.

**Figure 6:**
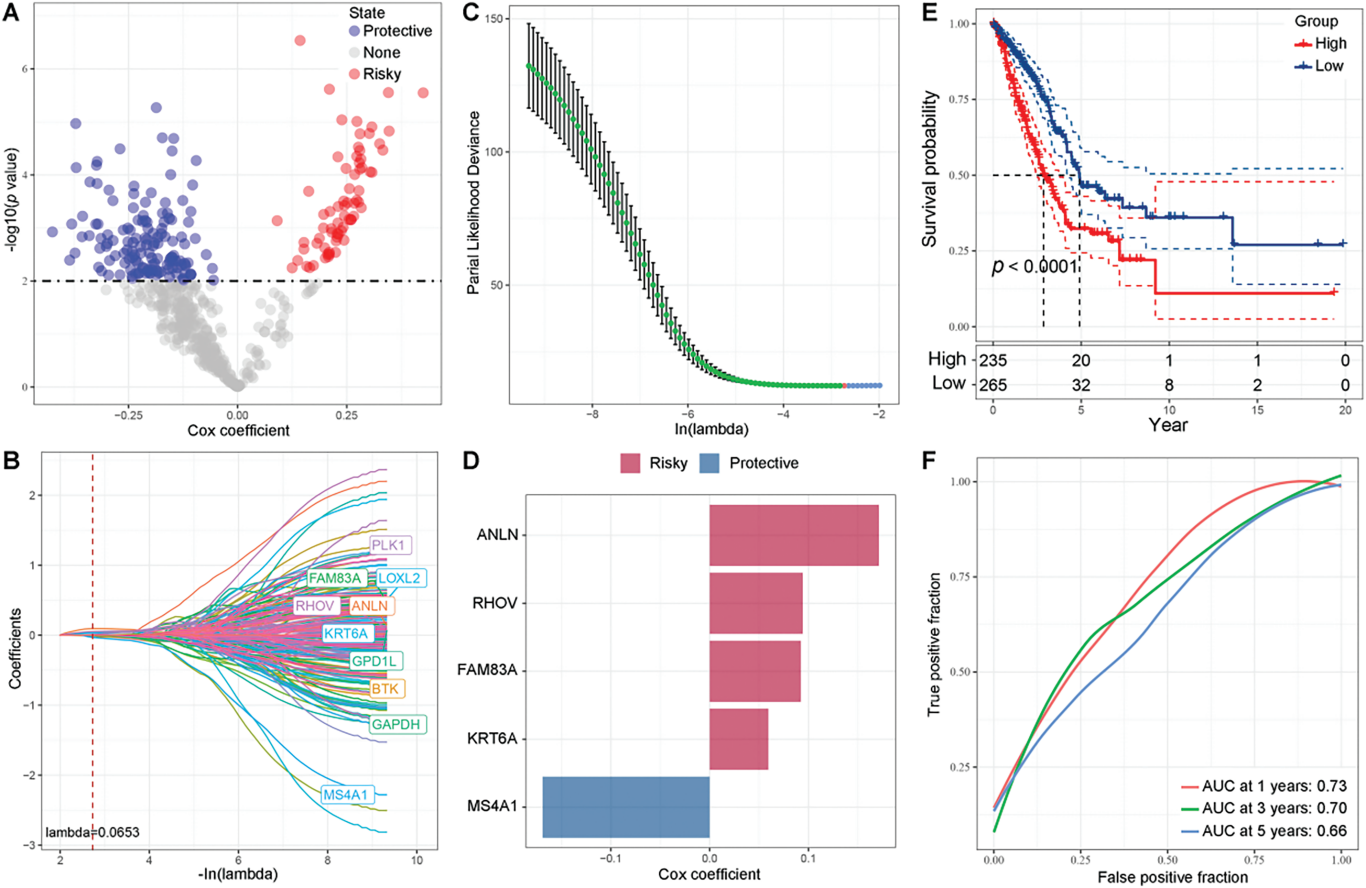
Prognostic gene identification and risk assessment. (**A**) Volcano plot depicting the results of univariate Cox regression analysis, with red representing risky genes, blue representing protective genes, and gray representing genes with no significant difference. (**B,C**) Coefficient distribution diagram of each gene and optimal λ for constructing LASSO model. (**D**) Cox coefficient of the five prognostic genes. (**E**) Kaplan-Meier analysis results of high- and low-risk groups based on the TCGA-LUAD cohort. (**F**) ROC analysis based on the TCGA-LUAD cohort

From univariate Cox regression analysis, we identified 140 protective genes and 73 risky genes (Fig. 6A). A five-gene prognostic signature was successfully formulated through LASSO regression analysis, with the optimal λ identified as 0.0653 via ten-fold cross-validation (Fig. 6B,D). Risk score = 0.171 ∗ ANLN + 0.092 ∗ FAM83A + 0.094 ∗ RHOV + 0.059 ∗ KRT6A − 0.169 ∗ MS4A1. Worse prognosis of patients with high risk score were observed compared to counterparts with low risk score (*p* < 0.0001, Fig. 6E). The AUCs for 1-, 3-, and 5-year survival were 0.73, 0.70, and 0.66, respectively (Fig. 6F). Survival probabilities were well stratified across all five internal validation cohorts (*p* < 0.0047, *p* = 0.0001, *p* = 0.018, *p* = 0.0066, and *p* = 0.0001, Supplementary Fig. S3A–E). The AUCs for 1-, 3-, and 5-year survival further validated the robustness of the prognostic signature across all five internal validation cohorts (Supplementary Fig. S3A–E).

### 3.6 A Predictive Nomogram Was Established Based on Risk Score and Clinicopathological Features

**Figure 7:**
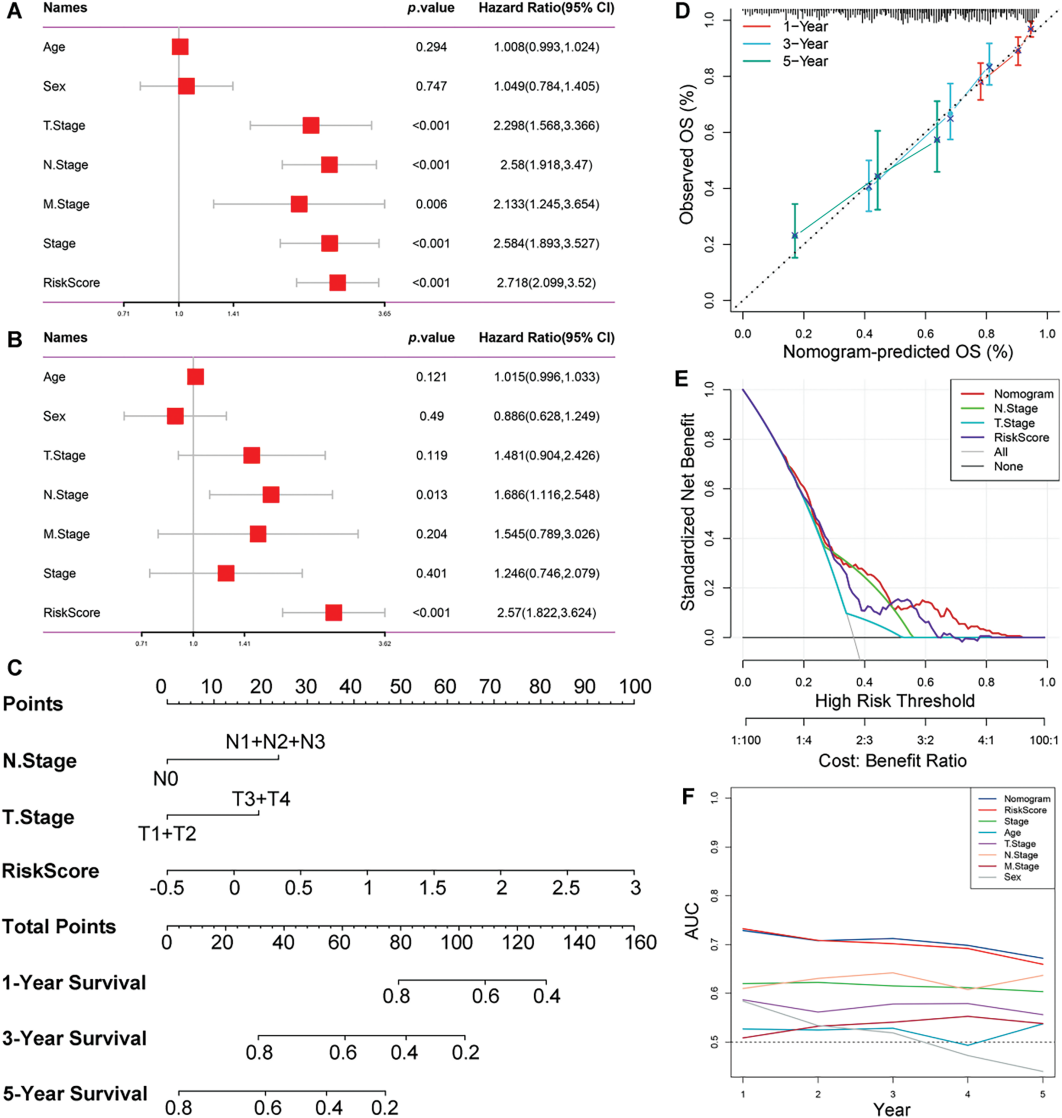
Comprehensive evaluation and validation of prognostic nomogram. (**A,B**) Univariate and multivariate Cox analyses assessing the independence of the predictive risk models. (**C**) Prognostic nomogram combining risk score and clinical characteristics. (**D**) Calibration curves demonstrating the predictable capability of the nomogram at 1-, 3-, and 5-year. (**E**) Standardized net benefit decision curves. (**F**) AUCs of the nomogram, risk score, and other clinical characteristics

We observed significant differences in risk scores among patients characterized by various clinicopathological features (Supplementary Fig. S4A–F). Both N stage (*p* = 0.013, HR = 1.686 [1.116–2.548]) and risk score (*p* < 0.001, HR = 2.57 [1.822–3.624]) were identified as independent prognostic factors for LUAD through univariate and multivariate Cox regression analyses (Fig. 7A,B). Risk score and other clinicopathological features were further integrated to construct a predictive nomogram for predicting survival outcomes (Fig. 7C). The calibration curve demonstrated that the predicted survival probabilities at 1-, 3-, and 5-year intervals closely aligned with the ideal line (Fig. 7D). Furthermore, the decision curve analysis indicated that the nomogram exhibited optimal clinical benefit across varying threshold probabilities (Fig. 7E). ROC analyses further confirmed the predictive efficacy of the nomogram (Fig. 7F).

### 3.7 Functional Associations of the Five Genes in the Prognostic Signature

Functional characterization revealed that the five prognostic genes were strongly correlated with immune response pathways. Specifically, Membrane spanning 4 domains A1 (MS4A1) and RHOV showed significant positive correlations with immune-related pathways (Supplementary Fig. S5A). The functional annotation was quantified for each TCGA-LUAD sample and represented as a distribution heat map (Supplementary Fig. S5B).

### 3.8 Immunological Dissection between Different Risk Groups

**Figure 8:**
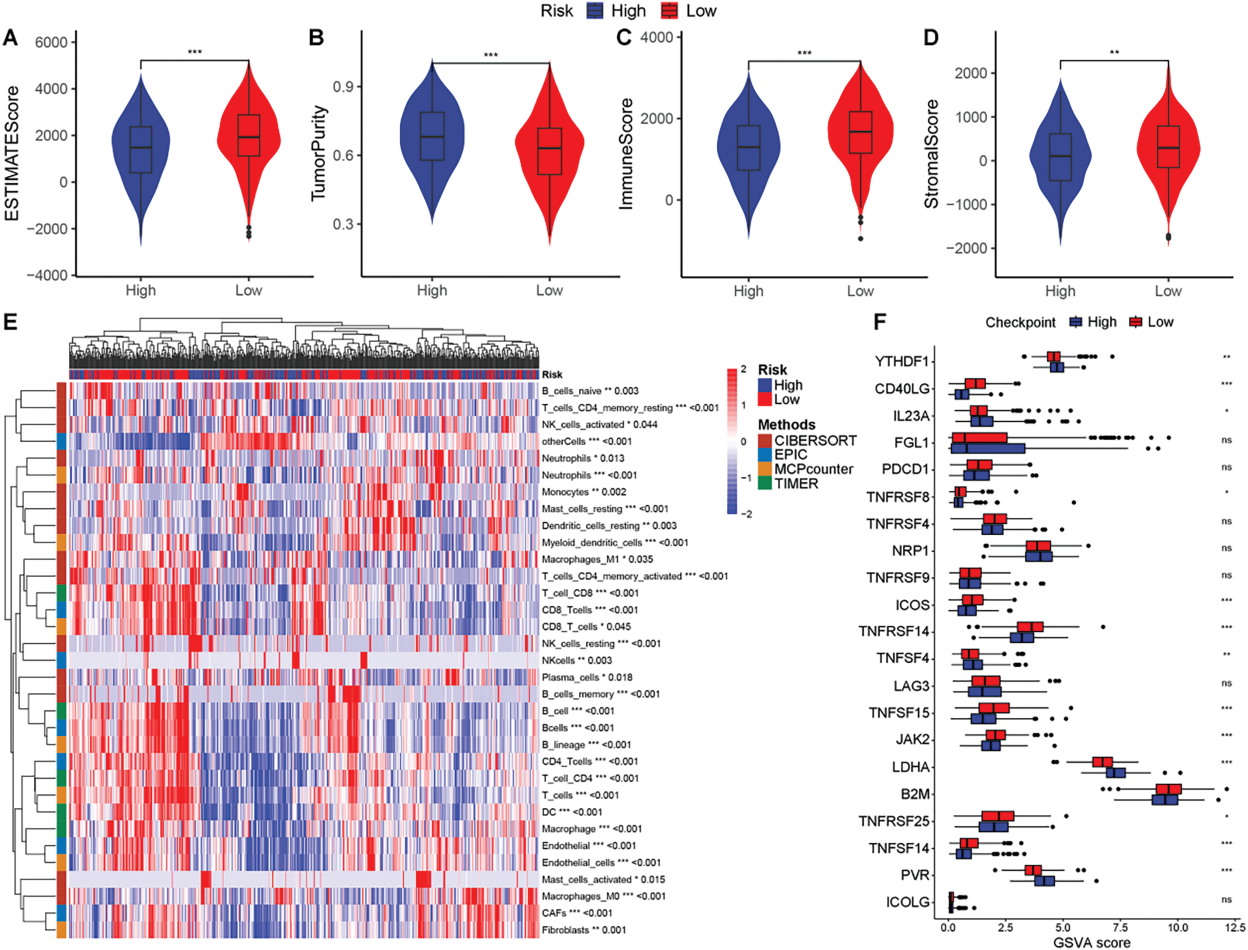
Correlation of prognostic genes with immune and stromal components. (**A–D**) Comparison of ESTIMATE scores, tumor purity, immune scores, and stromal scores between different risk groups. (**E**) Differences in the abundance of immune infiltrating cells between high and low-risk groups. (**F**) Comparison of immune checkpoint scores between high and low-risk groups (*p < 0.05, **p < 0.01, ***p < 0.001, ns indicates non-significant)

Analysis revealed that in the TME of high-risk group, stromal and immune components were more lower, while tumor component was more higher (Fig. 8A–D). The complex heatmap illustrated the differential infiltration of multiple immune cells across risk groups (Fig. 8E). Nearly all immune cells exhibited differential enrichment in the TME of the risk groups. Additionally, a majority of immune checkpoints were significantly elevated in the low-risk group (Fig. 8F).

### 3.9 The Prognostic Signature Was Capable of Predicting the Immunotherapy Response

**Figure 9:**
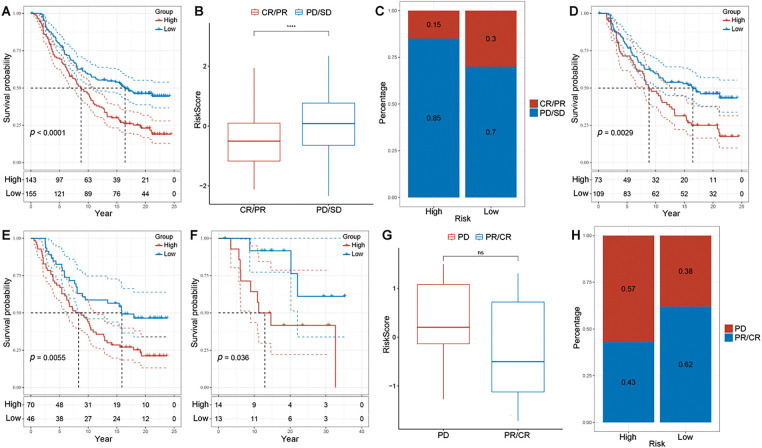
Evaluating the risk model’s predictive accuracy for treatment response and survival. (**A**) Kaplan-Meier analysis based on IMvigor210 dataset. (**B**) Differences in risk scores for different clinical response groups based on the IMvigor210 dataset. (**C**) Proportions of high- and low-risk patients in different clinical response groups based on the IMvigor210 dataset. (**D**) Kaplan-Meier analysis comparing patients in clinical stages I–II with different risk scores based on the IMvigor210 dataset. (**E**) Kaplan-Meier analysis comparing patients in clinical stages III-IV with different risk scores based on the IMvigor210 dataset. (**F**) Kaplan-Meier analysis based on the GSE78220 dataset. (**G**) Differences in risk scores for different clinical response groups based on the GSE78220 dataset. (**H**) Proportions of high- and low-risk patients in different clinical response groups based on the GSE78220 dataset (****p < 0.0001, ns indicates non-significant)

We divided patients from the IMvigor210 and GSE78220 cohorts into high- and low-risk groups. Patients in high-risk group exhibited worse prognosis (IMvigor210: *p* < 0.0001; GSE78220: *p* = 0.036, Fig. 9A,F). In the IMvigor210 cohort, the overall risk scores of patients with complete response (CR) or partial response (PR) were significantly lower compared to those with progressive disease (PD) and stable disease (SD) (Fig. 9B). The percentage of patients with PD/SD was also higher in the high-risk group compared to the low-risk group (85% *vs*. 70%, Fig. 9C). Besides, the predictive risk model was more sensitive in forecasting the prognosis of patients with early-stage disease (Fig. 9D,E). In the GSE78220 cohort, no significant difference in risk scores was observed between patients with PD and those with CR/PR (Fig. 9G). The percentage of patients with PD was significantly higher in the high-risk group compared to the low-risk group (57% *vs*. 38%, Fig. 9H). These results indicate that the prognostic signature effectively predicts the immunotherapy response in patients with LUAD.

### 3.10 RHOV Was Crucial for the Growth of Lung Epithelial Cells

**Figure 10:**
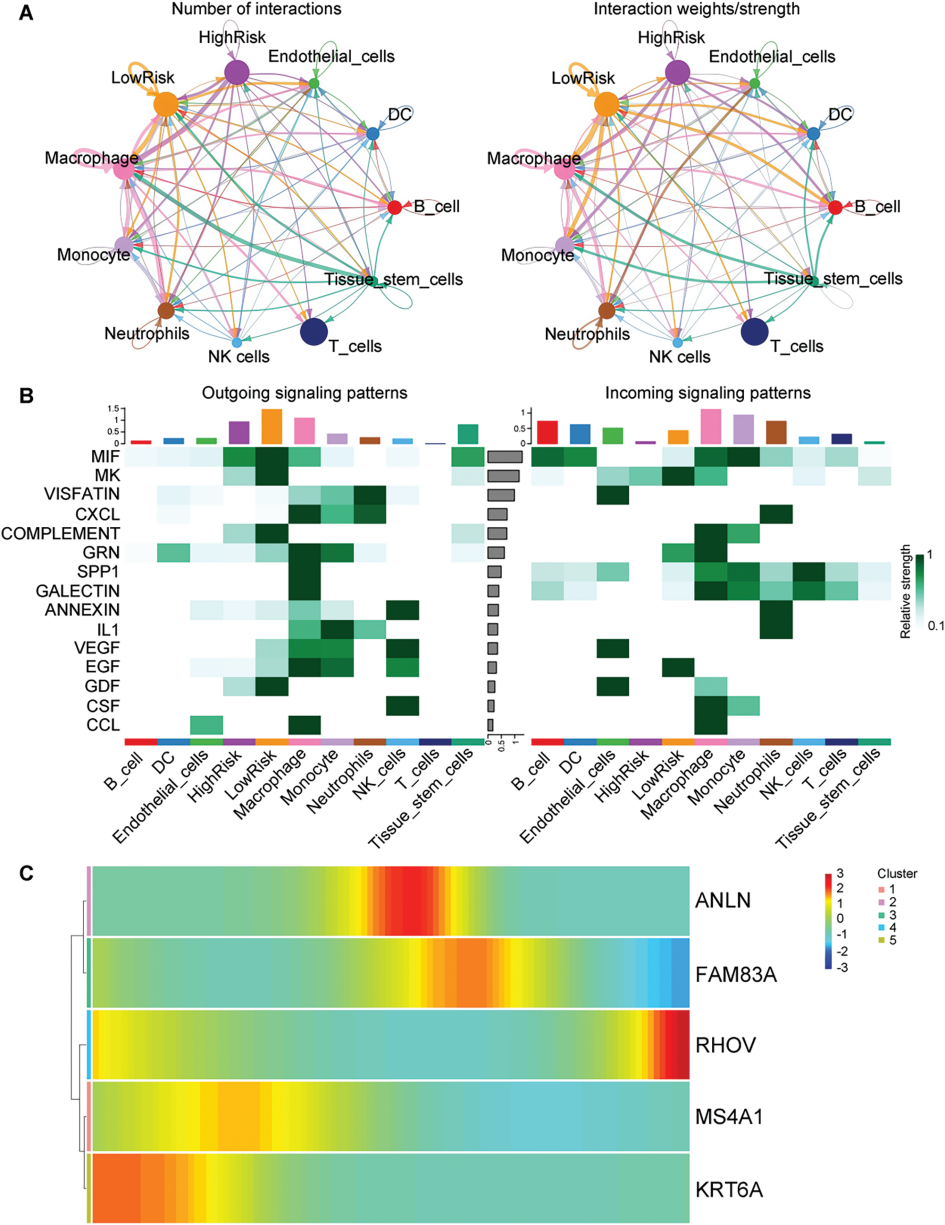
Cell type interaction and trajectory analysis in KRAS/TP53 MUT and WT groups. (**A**) Number of interactions between cells and the strength of these interactions. (**B**) Heatmap depicting the signal flow pattern of mutual recognition among cells in the KRAS/TP53 MUT group. (**C**) Heatmap showing the expression of seven prognostic genes over pseudotime

To further investigate the five prognostic genes, we scored each gene in MUT LUAD and WT LUAD using five scoring algorithms. The total score (Scoring) for each cell was calculated as the sum of these scoring algorithms (Supplementary Fig. S6A). The results revealed significant differences in Scoring among several cell types between MUT LUAD and WT LUAD (Supplementary Fig. S6B). Notably, the Scoring of epithelial cells was significantly higher in MUT LUAD compared to WT LUAD. Given that LUAD derives from the bronchial mucosa, we focused on epithelial cells for subsequent analyses. HighRisk and LowRisk epithelial cells were classified and distinct intercellular communication pattern was observed between these two subtypes (Fig. 10A). Stronger signaling pattern was determined in LowRisk epithelial cells compared to HighRisk ones (Fig. 10B). Additionally, as the trajectory evolved, RHOV expression was gradually upregulated, while Keratin 6A (KRT6A) expression was gradually downregulated (Supplementary Fig. S6C, Fig. 10C).

### 3.11 Downregulating RHOV Significantly Suppressed Tumorigenesis

**Figure 11:**
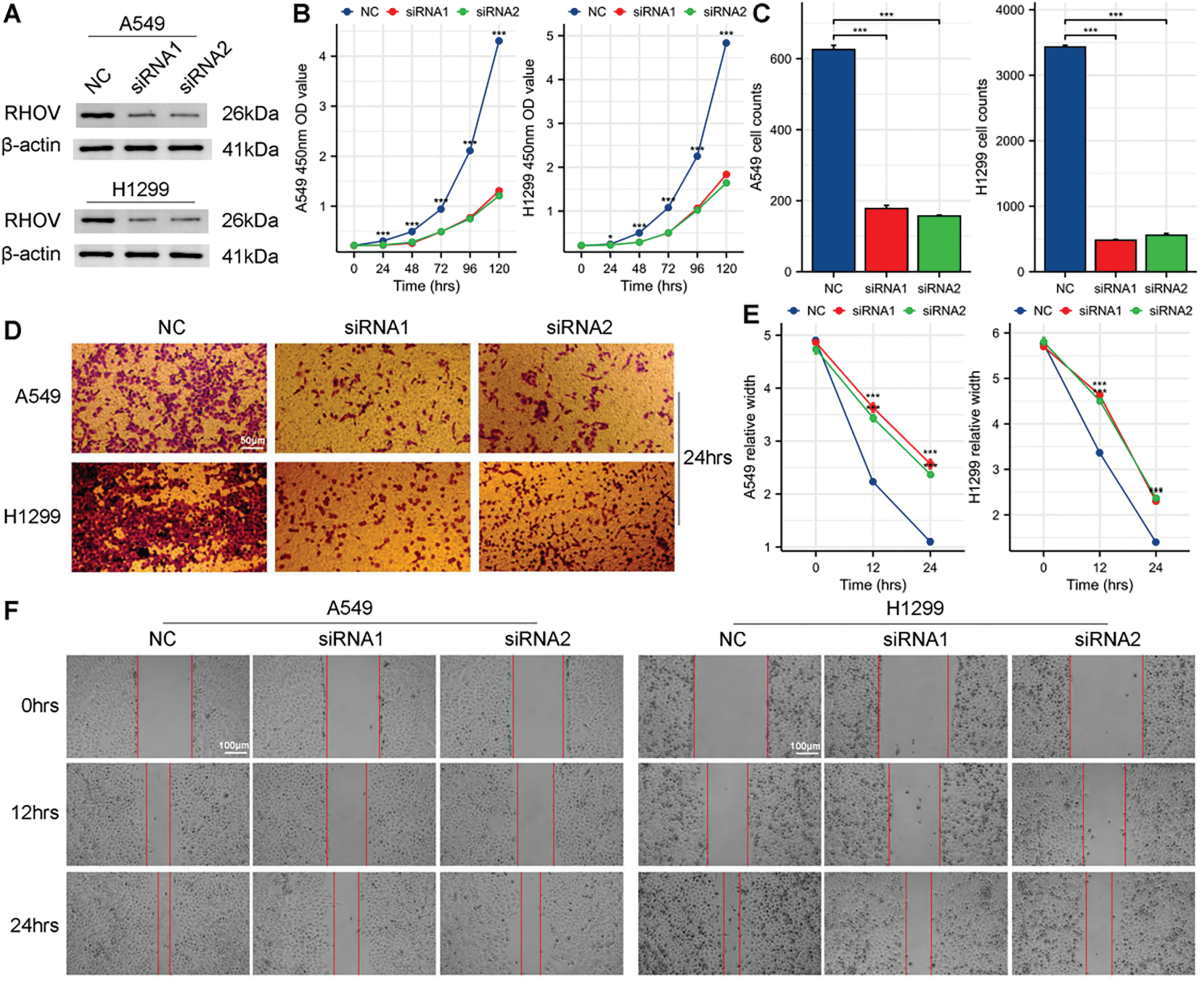
Impact of RHOV knockdown on protein levels, proliferation, invasion, and migration in A549 and H1299 Cells. (**A**) Western blot assay detecting RHOV expression in A549 and H1299 cells after treatment with siRNAs. (**B**) CCK8 assay assessing the proliferation ability of A549 and H1299 cells following siRNA treatment (**C,D**) Transwell assay evaluating the migration ability of A549 and H1299 cells after siRNA treatment. (**E,F**) Wound healing assay assessing the invasion ability of A549 and H1299 cells following siRNA treatment (*p < 0.05, ***p < 0.001)

Given the progressively elevated expression of RHOV during the evolution trajectory of lung epithelial cells, subsequent functional experiments focused on RHOV. Western blot assay confirmed that both siRNA1 and siRNA2 effectively reduced RHOV protein expression (Fig. 11A). CCK8 assay demonstrated that silencing RHOV impaired the proliferative capabilities of A549 and H1299 cells (Fig. 11B). Consistent results from transwell and wound healing assays indicated decreased capabilities of migration and invasion in A549 and H1299 cells upon downregulation of RHOV (Fig. 11C–F). These findings provided compelling evidence for RHOV as a pivotal gene during LUAD progression.

## 4 Discussion

Tumor heterogeneity encompasses not only the differences within cell subclones but also the diverse composition of the TME, which significantly influences the evolutionary fate of cancer cells. Gene mutations serve as critical upstream modulators of cancer progression. However, the predominant effect of genetic heterogeneity on TME dynamics requires more elucidation. Our investigation systematically dissected the characteristics of the TME in LUAD with KRAS/TP53 mutations, leading to the development of a prognostic stratification method and the possibility of a therapeutic target.

Neutrophils act as primary sentinels in innate anti-cancer immune responses, yet they can be co-opted by cancer cells to promote carcinogenesis [18,19]. Unraveling the interactions between neutrophils and tumor cells may enhance our understanding of cancer etiology and inform novel therapeutic strategies. Notably, we identified a higher proportion of neutrophils within the TME of KRAS/TP53-mutated LUAD, leading to the identification of two novel neutrophil subtypes: IS MUT and WT neutrophils. The IS MUT neutrophils were found to be more abundant in LUAD with KRAS/TP53 mutations compared to WT LUAD. Intercellular communication analysis indicated a more active signaling network among IS MUT neutrophils. These findings suggest a causal relationship between KRAS/TP53 mutations and neutrophil heterogeneity in LUAD, indicating that genomic mutations may drive TME heterogeneity during tumor evolution. Our findings align with prior studies demonstrating the prognostic value of neutrophil counts and neutrophil-to-lymphocyte ratio (NLR) in NSCLC [20–22]. However, while peripheral neutrophil counts reflect systemic inflammation, our study highlights the unique prognostic significance of tumor-infiltrating neutrophil subpopulations (e.g., IS MUT) driven by KRAS/TP53 mutation. This suggests that TME-specific neutrophil heterogeneity may serve as a more precise biomarker for LUAD prognosis than traditional hematological parameters, potentially guiding personalized therapeutic strategies such as targeting RHOV or modulating IS MUT neutrophil activity. He et al. [23] also highlighted TME heterogeneity driven by Epidermal growth factor receptor (EGFR) mutations in LUAD, revealing stromal cell heterogeneity and complex intercellular interactions. They found significant enrichment of Cluster of differentiation 1C (CD1C)^+^ dendritic cells, with infiltrating T cells primarily exhibiting exhausted and regulatory characteristics within tumors. E74-like ETS transcription factor 3 (ELF3) was identified as a key target promoting LUAD progression through pseudotime trajectory analysis. Thus, TME heterogeneity reflects genomic diversity, including gene mutations, at a single-cell resolution. Our study extends this paradigm by linking KRAS/TP53 mutations to neutrophil reprogramming, thereby bridging genetic alterations with immune microenvironment remodeling—a critical gap in current literature. Subsequently, we identified 70 candidate genes associated with IS MUT neutrophils through hdWGCNA and univariate Cox regression analysis. Among these, RHOV was found to accumulate in lung epithelial cells as pseudo time progressed, and its downregulation attenuated lung cancer progression *in vitro*. This suggests a potential positive feedback loop where KRAS/TP53 mutations reshape the TME of LUAD, inducing the enrichment of IS MUT neutrophils during tumor evolution, while RHOV, as an IS MUT neutrophil-related gene, conversely contributes to tumor progression. This dual role of neutrophils—both as microenvironment modulators and tumor progression drivers—underscores their therapeutic potential. Clinically, monitoring IS MUT neutrophil abundance (e.g., via liquid biopsy or imaging-based TME profiling) could complement existing biomarkers like NLR to refine risk stratification. Furthermore, targeting RHOV or its downstream pathways may synergize with immune checkpoint inhibitors, particularly in KRAS/TP53-mutated LUAD patients with high IS MUT neutrophil infiltration.

We successfully established a prognostic signature containing five genes through LASSO regression analysis, which was validated for its predictive capability. The survival probabilities of LUAD patients could be significantly stratified based on the risk score. Moreover, significant differences in risk scores were observed across various clinicopathological features, including sex, T stage, N stage, and clinical stage. Cox regression analyses verified the independently prognostic effect of risk score. To further enhance the predictive performance of the prognostic signature, a nomogram with combination of risk score and clinicopathological features was generated to predict OS in LUAD patients. Although the increase in the AUC was modest, the inclusion of the nomogram significantly improved its clinical utility. The nomogram provided a comprehensive assessment of patient prognosis by integrating multiple clinicopathological features with the risk score, allowing for personalized treatment decisions in the clinically heterogeneous landscape of LUAD. Decision curve analysis further validated the nomogram’s utility in clinical decision-making, underscoring its value beyond mere numerical AUC enhancement.

Multiple previous studies have highlighted the cancerous roles of five prognostic genes: **MS4A1**, Actin binding protein (ANLN), Family with sequence similarity 83 member A (FAM83A), **RHOV**, and **KRT6A**. MS4A1, or CD20, is a molecule expressed on the surface of B cells that regulates their development and differentiation into plasma cells [24]. Recent investigations have reported the prognostic implications of MS4A1 in LUAD. Cong et al. [25] identified MS4A1 as a key gene associated with fatty acid metabolism, suggesting it plays a protective role in influencing LUAD prognosis. Furthermore, MS4A1 has been implicated as a positive prognostic factor concerning tumor microenvironment, tumor invasion, and immunological responses [26–28]. **ANLN** is involved in cell growth, migration, and cytokinesis, regulating actin cytoskeletal dynamics in podocytes, a component of the glomerulus [29]. Research has uncovered the tumor-promoting effects of ANLN in LUAD, indicating its overexpression correlates with a poorer prognosis, potentially due to enhanced tumor metastasis [30,31]. Moreover, ANLN’s role in LUAD may also be associated with preventing pyroptosis in cancer cells [32]. Notably, ANLN has been proposed as a therapeutic target, particularly through its interaction with Kaempferol, a potential ANLN inhibitor [33]. **FAM83A** is a critical participant in the cellular proliferative signaling network. Various studies suggest its oncogenic role in LUAD, concluding that increased levels of FAM83A are indicative of a negative prognosis and poorer clinical outcomes [34–38]. Mechanistic investigations have revealed that the oncogenic proliferation of lung cancer cells induced by FAM83A upregulation is primarily attributed to the aberrant activation of several growth-related signaling pathways, including Hippo, Wnt, Extracellular regulated protein kinase (ERK), and PI3K/Protein kinase B (AKT)/Mammalian target of rapamycin (mTOR) signaling pathways [39–41]. Additionally, Zhou et al. [42] demonstrated that overexpressed FAM83A is correlated with enhanced epithelial-mesenchymal transition (EMT), mediated by the ERK signaling pathway. RHOV has been identified as a negative prognostic factor for LUAD across multiple studies, emphasizing its potential as a therapeutic target [43–46]. Chen et al. [47] found that silencing RHOV significantly reduced the proliferation, migration, invasion, and tumorigenicity of LUAD cells. Subsequent *in vivo* experiments suggested that RHOV silencing increased gefitinib sensitivity in resistant LUAD cells and enhanced gefitinib-induced apoptosis. Zhang et al. [48] further reported that RHOV-overexpression-driven cell growth and metastasis could be inhibited by pyrazolanthrone, a c-Jun N-terminal kinase (JNK) inhibitor. KRT6A plays a role in the activation of follicular keratinocytes post-wounding and has been recognized as a prognostic indicator for patients with LUAD [49–51]. Both *in vivo* and *in vitro* analyses confirm that the progression of LUAD can be attenuated by eliminating KRT6A [52–54].

This study presents several strengths. First, the integration of single-cell and bulk transcriptomic data allows for a comprehensive exploration of TME heterogeneity, providing novel insights into neutrophil subpopulations. Second, the functional validation of RHOV through *in vitro* experiments strengthens the translational relevance of our findings. Third, the prognostic signature was rigorously validated across multiple independent cohorts, ensuring robustness. However, limitations exist. The retrospective nature of public datasets may introduce selection bias, and the absence of prospective validation cohorts limits the immediate clinical applicability of our results. Additionally, while IS MUT neutrophils were identified as key players, their functional interactions with other immune cells remain unexplored. Future studies should aim to validate these findings in larger, ethnically diverse cohorts and investigate therapeutic strategies targeting RHOV or IS MUT neutrophils in preclinical models.

## 5 Conclusion

This study primarily elucidates the potential effects of KRAS/TP53 mutations on neutrophil heterogeneity in the TME of LUAD. These mutations may influence prognosis through the regulation of neutrophil subpopulations. Furthermore, the neutrophil-associated prognostic signature provides novel insights into prognosis prediction and clinical decision-making. Importantly, RHOV, as a pivotal proliferative factor for lung epithelial cells, holds promise as therapeutic prioritization for mutation-specific LUAD.

## Supplementary Materials

Supplementary Figure S1 Comparative analysis of immune infiltration and functionality between clusters. A. Degree of immune cell infiltration across different clusters. B. Comparison of ImmuneScore, StromalScore, and ESTIMATEScore between clusters. C. Expression levels of immune-related genes across clusters. D-I. Differences in TIDE, IFNG, dysfunction score, exclusion score, and proportions of TAM M2 and MDSC between clusters. (*p < 0.05, **p < 0.01, ***p < 0.001, ****p < 0.0001, ns indicates non-significant)

Supplementary Figure S2 Differential gene expression and functional enrichment analysis. A. Volcano plot illustrating the results of differential expression analysis, with blue representing down-regulated DEGs, red representing up-regulated DEGs, and gray indicating genes with no significant difference. B. Results from GO enrichment analysis. C. Results from KEGG enrichment analysis.

Supplementary Figure S3 Validation of prognostic analysis across multiple external datasets. A-E. Kaplan-Meier analyses and corresponding ROC analysis results based on the GSE68465, GSE3141, GSE31210, GSE37745, and GSE50081 datasets.

Supplementary Figure S4 Risk score distribution across various clinical subgroups. A-F. Box plots displaying risk scores across various age groups, TNM stages, tumor stages, and sex. (*p < 0.05)

Supplementary Figure S5 Correlation of prognostic genes with pathways and functional enrichment in LUAD. A. Heatmap showing correlations between prognostic genes and signature pathways based on the TCGA-LUAD dataset. B. Heatmap illustrating the expression of prognostic genes and functional enrichment for each tumor sample based on the TCGA-LUAD dataset. (*p < 0.05, **p < 0.01, ***p < 0.001)

Supplementary Figure S6 Cell type scoring and trajectory analysis in KRAS/TP53 MUT and WT groups. A. Violin plot displaying the prognostic gene set scores for ten cell types in the KRAS/TP53 MUT and WT groups based on different algorithms. B. Violin plot showing the scoring of ten cell types on the prognostic gene set between the KRAS/TP53 MUT and WT groups. C. Trajectories illustrating pseudo-time-dependent cellular states of epithelial cells in the KRAS/TP53 MUT group. (**p < 0.01, ****p < 0.0001, ns indicates non-significant)

## Data Availability

This research utilized openly accessible data, which is accessible in the appropriate databases.

## Supplementary Materials

The supplementary material is available online at https://www.techscience.com/doi/10.32604/or.2025.062584/s1.
