# Supplementary figures and images for: Single-Cell Transcriptomic Profiling Reveals KRAS/TP53-Driven Neutrophil Reprogramming in Luad: A Multi-Gene Prognostic Model and Therapeutic Targeting of RHOV
Source: Oncol Res. 2025 May 29;33(6):1383–404. doi: 10.32604/or.2025.062584 (PMC12144629; doi:10.32604/or.2025.062584)

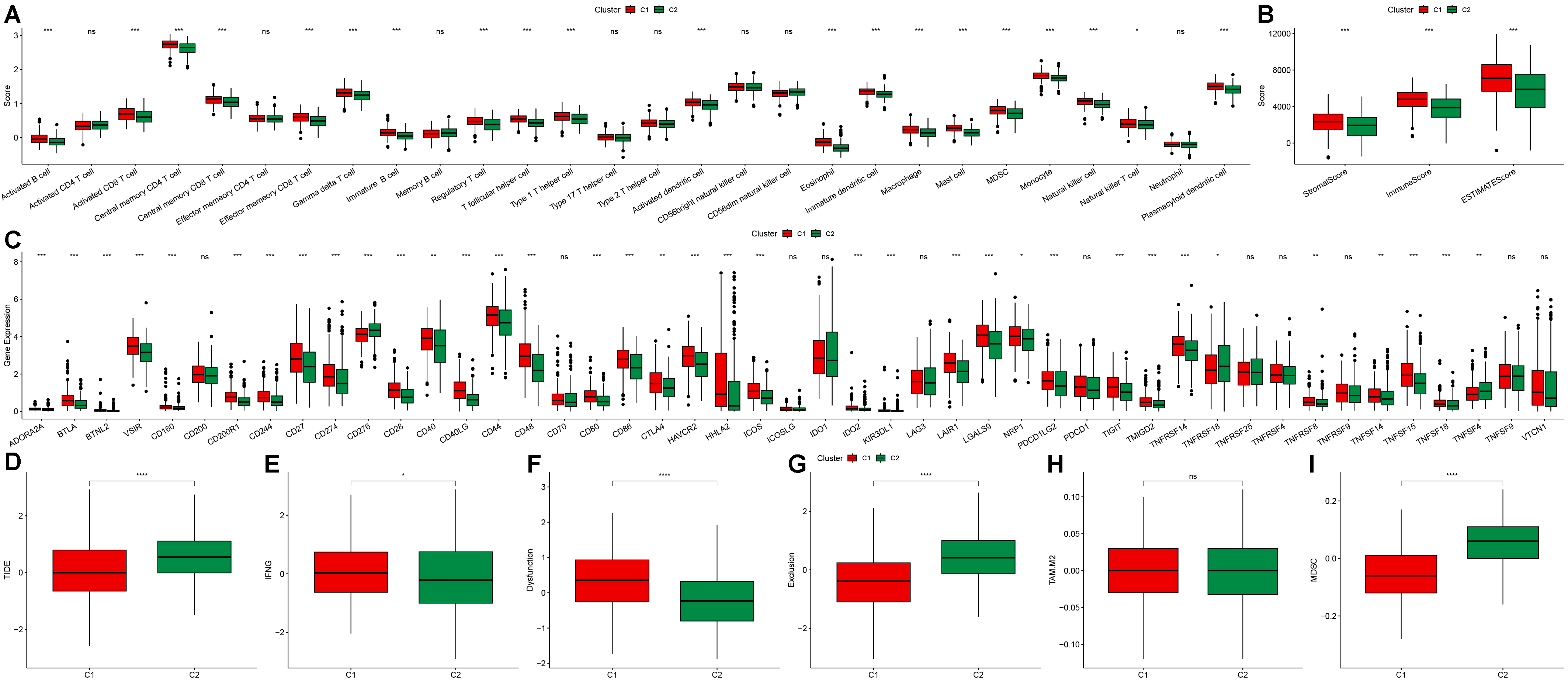

Supplement: Supplementary Figure S1 — A. Degree of immune cell infiltration across different clusters. B. Comparison of ImmuneScore, StromalScore, and ESTIMATEScore between clusters. C. Expression levels of immune-related genes across clusters. D-I. Differences in TIDE, IFNG, dysfunction score, exclusion score, and proportions of TAM M2 and MDSC between clusters. (*p < 0.05, **p < 0.01, ***p < 0.001, ****p < 0.0001, ns indicates non-significant) [file OncolRes-33-62584-s001.tif]

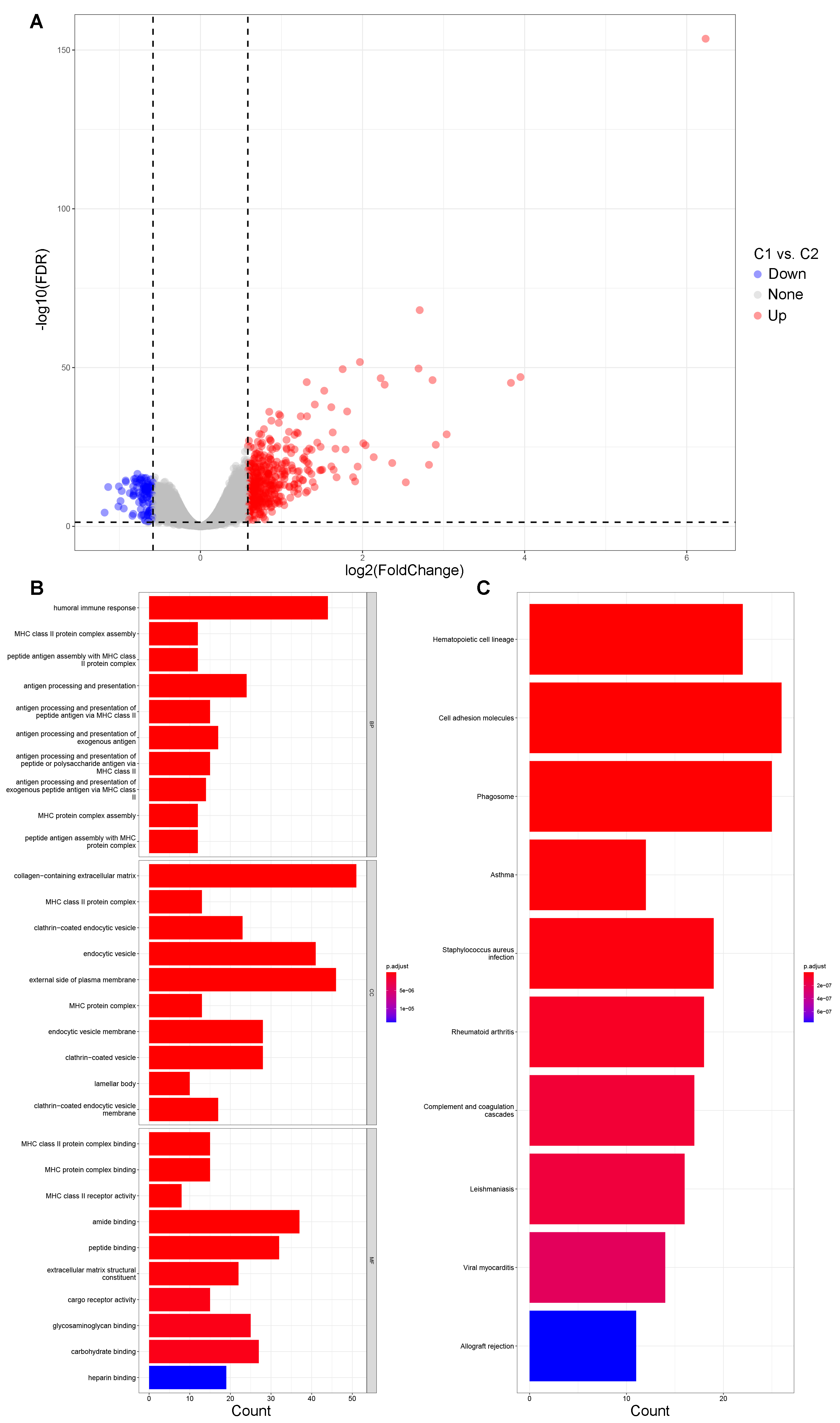

Supplement: Supplementary Figure S2 — A. Volcano plot illustrating the results of differential expression analysis, with blue representing down-regulated DEGs, red representing up-regulated DEGs, and gray indicating genes with no significant difference. B. Results from GO enrichment analysis. C. Results from KEGG enrichment analysis. [file OncolRes-33-62584-s002.tif]

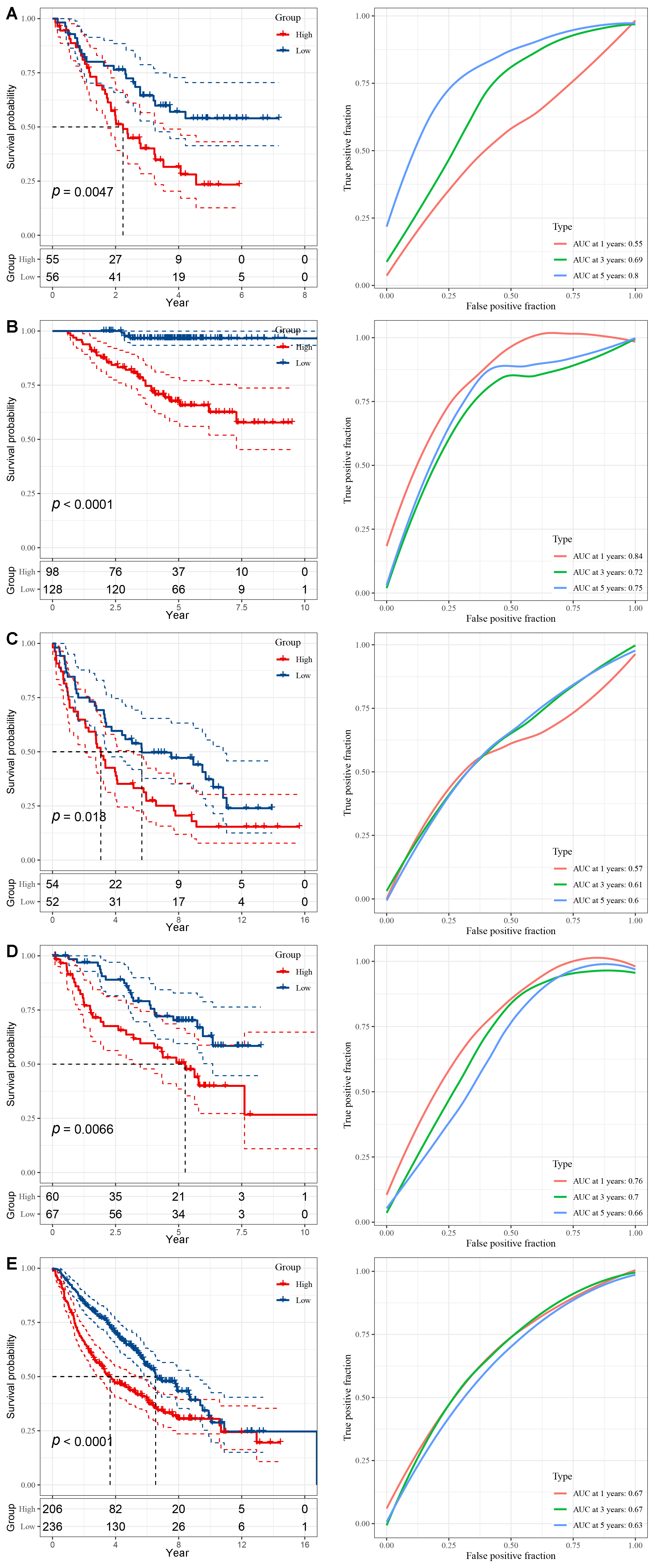

Supplement: Supplementary Figure S3 — A-E. Kaplan-Meier analyses and corresponding ROC analysis results based on the GSE68465, GSE3141, GSE31210, GSE37745, and GSE50081 datasets. [file OncolRes-33-62584-s003.tif]

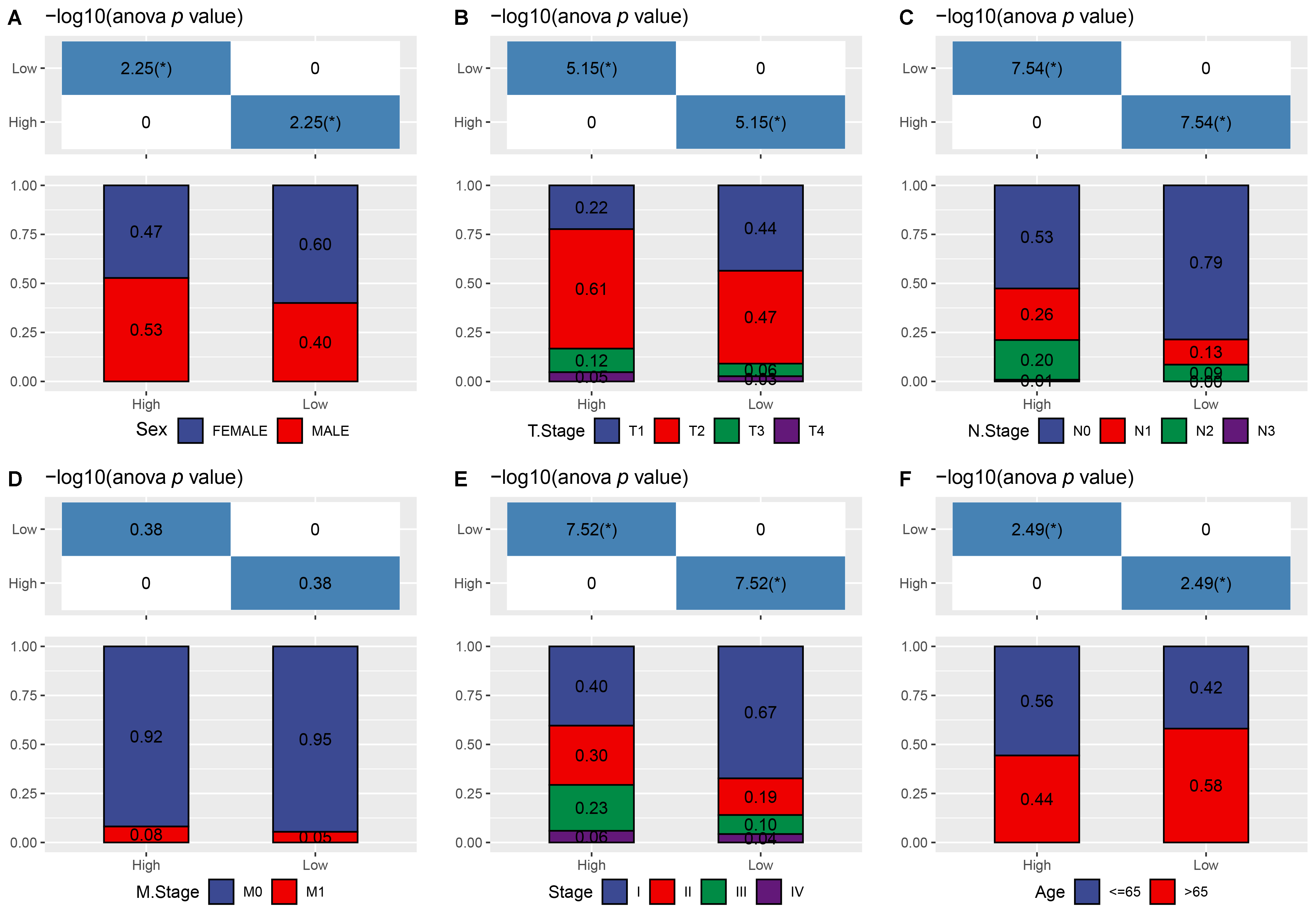

Supplement: Supplementary Figure S4 — A-F. Box plots displaying risk scores across various age groups, TNM stages, tumor stages, and sex. (*p < 0.05) [file OncolRes-33-62584-s004.tif]

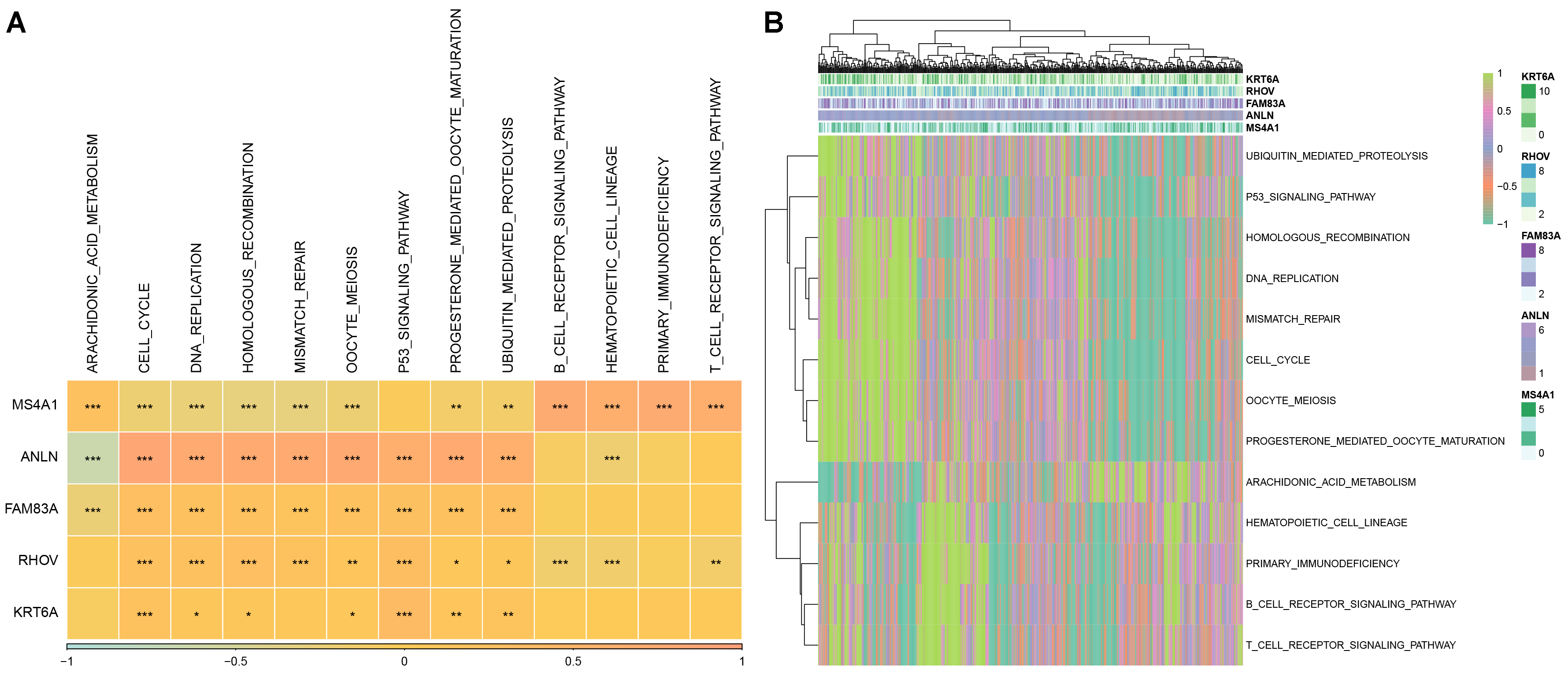

Supplement: Supplementary Figure S5 — A. Heatmap showing correlations between prognostic genes and signature pathways based on the TCGA-LUAD dataset. B. Heatmap illustrating the expression of prognostic genes and functional enrichment for each tumor sample based on the TCGA-LUAD dataset. (*p < 0.05, **p < 0.01, ***p < 0.001) [file OncolRes-33-62584-s005.tif]

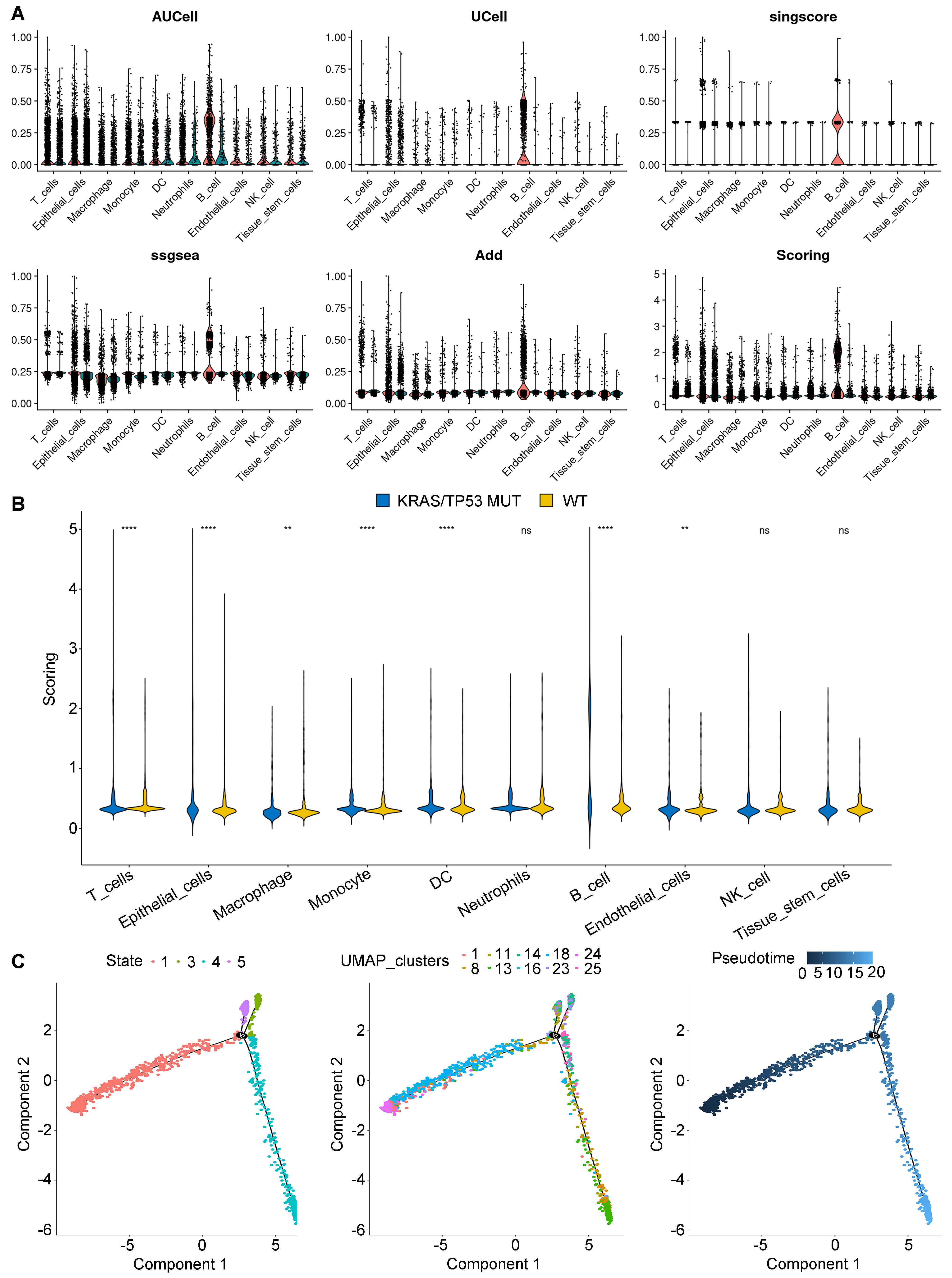

Supplement: Supplementary Figure S6 — A. Violin plot displaying the prognostic gene set scores for ten cell types in the KRAS/TP53 MUT and WT groups based on different algorithms. B. Violin plot showing the scoring of ten cell types on the prognostic gene set between the KRAS/TP53 MUT and WT groups. C. Trajectories illustrating pseudo-time-dependent cellular states of epithelial cells in the KRAS/TP53 MUT group. (**p < 0.01, ****p < 0.0001, ns indicates non-significant) [file OncolRes-33-62584-s006.tif]
